# Low Fluorodeoxyglucose Uptake in the Metastatic Lung Tumor From Clear Cell Renal Cell Carcinoma

**DOI:** 10.7759/cureus.67854

**Published:** 2024-08-26

**Authors:** Mami Yoshida, Shoji Oura

**Affiliations:** 1 Surgery, Kishiwada Tokushukai Hospital, Kishiwada, JPN

**Keywords:** metastatic lung tumor, low suv max, lipid, glycogen, clear cell renal cell carcinoma

## Abstract

A 78-year-old man had undergone laparoscopic left nephrectomy for clear cell renal cell carcinoma (CCRCC) and, three years later, partial resection for a lung metastasis of CCRCC. Follow-up computed tomography (CT) again showed a solitary oval lung nodule that was adjacent to the pulmonary vein, leading to careful CT follow-up without trans-bronchial lung biopsy. The lung nodule grew rapidly from 18 mm to 25 mm in a year. However, positron emission tomography showed only a slight increase in the maximal standardized uptake value (SUV max) of 3.76 g/mL. Under the tentative diagnosis of a metastatic lung tumor from CCRCC, the patient underwent lung wedge resection for the presumed lung metastasis. A postoperative pathological study showed a well-circumscribed oval tumor consisting of atypical cells with clear cytoplasm and densely growing in an expansive manner. Immunostaining using paraffin-embedded tissue showed that lipid droplets were observed on the tumor cells and periodic-acid Schiff (PAS)-positive granules were confirmed in the cytoplasm of the atypical cells. Oncologists should note that the SUV max value of metastatic lung tumors from CCRCC is ostensibly low due to the presence of intracytoplasmic lipids and glycogen.

## Introduction

The lung is an important organ along with the bone, liver, and brain as a site of hematogenous metastasis of solid malignancies. Metastatic lung tumors can be often cured by surgery [1,2] or radiation therapy [3,4] and therefore should be diagnosed as early as possible for better clinical outcomes.

Computed tomography (CT) plays a major role in the detection of pulmonary metastasis in the postoperative surveillance of various solid malignancies. Small lung lesions, however, are generally difficult to be judged as benign or malignant and often require repeat image evaluation after a certain period of time.

Positron emission tomography (PET) is effective in judging the target lesions, not only large tumors but also small tumors approximately 1 cm in size, whether to be benign or malignant by evaluating the glucose metabolism of the lesions [5]. In short, even if the pulmonary lesion is small, it is often possible to diagnose it as metastasis when fluorodeoxyglucose (FDG) uptake is avid.

We herein report a case of a metastatic lung tumor from clear cell renal cell carcinoma (CCRCC) in which the cause of a low maximal standard uptake (SUV max) value was pathologically clarified.

## Case presentation

**Figure 1:**
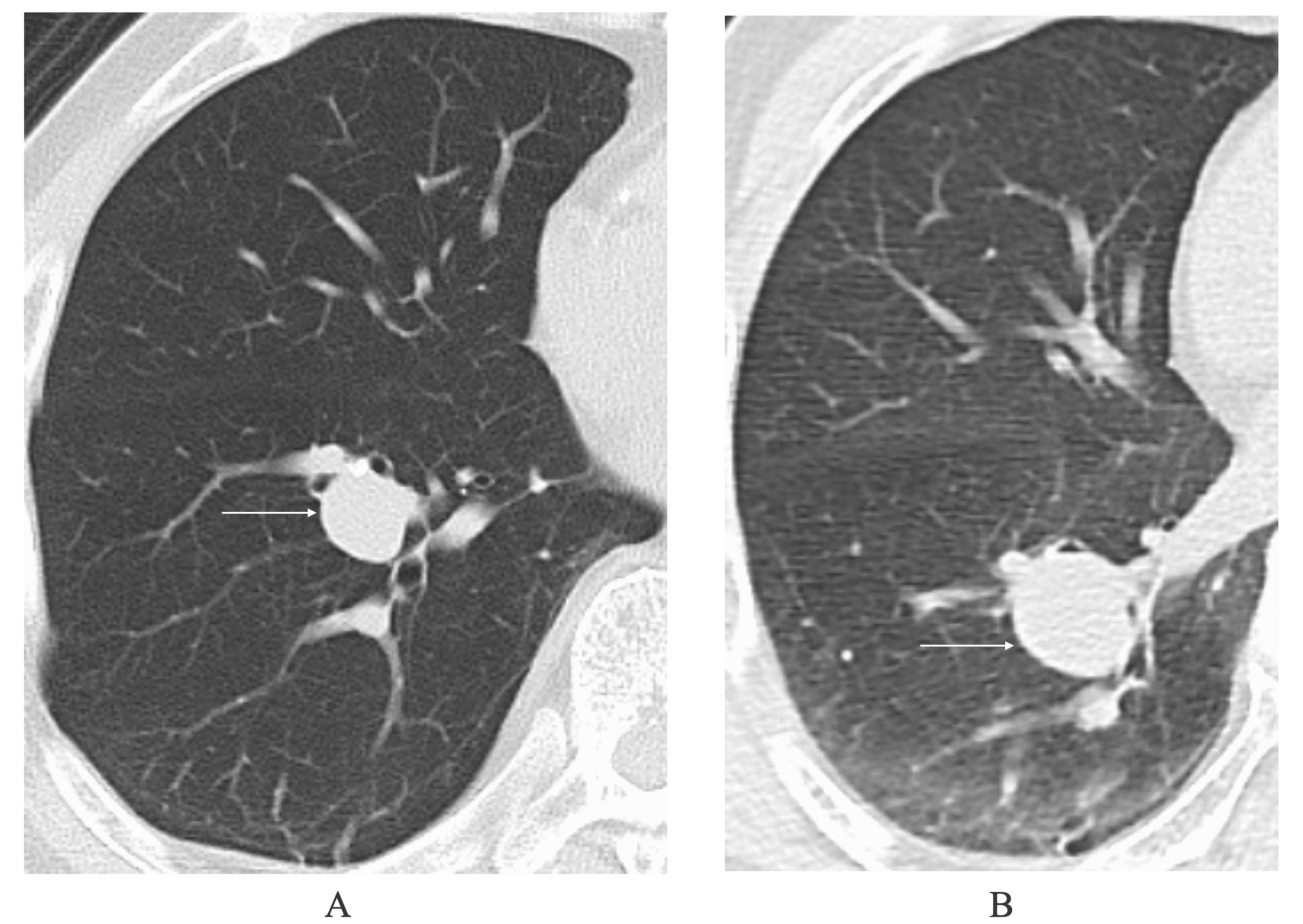
Computed tomography (CT) findings A. CT showed a pulmonary nodule (arrow) in close contact with the inferior pulmonary vein. B. One year observation caused growth of the pulmonary nodule (arrow).

A 78-year-old man had undergone laparoscopic left nephrectomy for CCRCC more than nine years before. Three years later, the patient had also developed a lung metastasis of the CCRCC and had received surgical treatment for it. The patient further showed a high prostate-specific antigen (PSA) level of 7.36 ng/mL and received PSA monitoring therapy [6] under the presumed diagnosis of cT1N0M0 prostate cancer. When the prostate cancer could be detected with magnetic resonance images, the patient was referred to our hospital for surgical treatment of the prostate cancer five years after the nephrectomy. Sixteen months after the prostate cancer surgery, CT again showed a small lung nodule in the right lower lobe (Figure 1A). Due to the location of the lung nodule in close contact with the inferior pulmonary vein, the patient received CT follow-up without receiving trans-bronchial lung biopsy. One year follow-up resulted in the tumor growth from 18 mm to 25 mm in size (Figure 1B).

**Figure 2:**
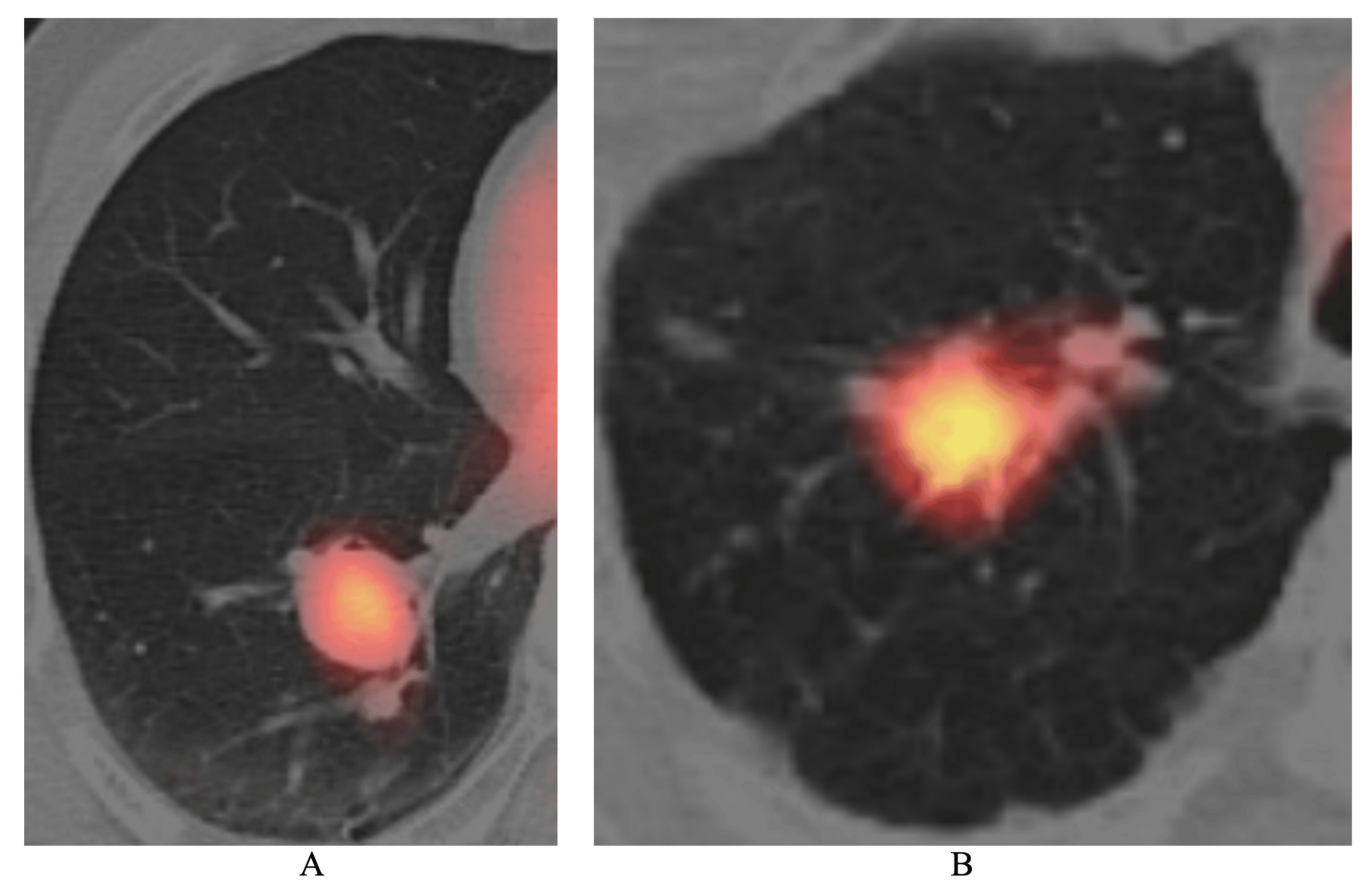
Positron emission tomography (PET) findings A. The maximal standardized uptake value (SUV max) of the tumor showed 3.76 g/mL just before operation. B. PET,  as a reference image, of the metastatic lung tumor from breast cancer showed a SUV max value of 10 g/mL despite the similar tumor size to this case.

However, PET of the lung lesion showed only a slight increase in the SUV max value of 3.76 g/mL (Figure 2A).

**Figure 3:**
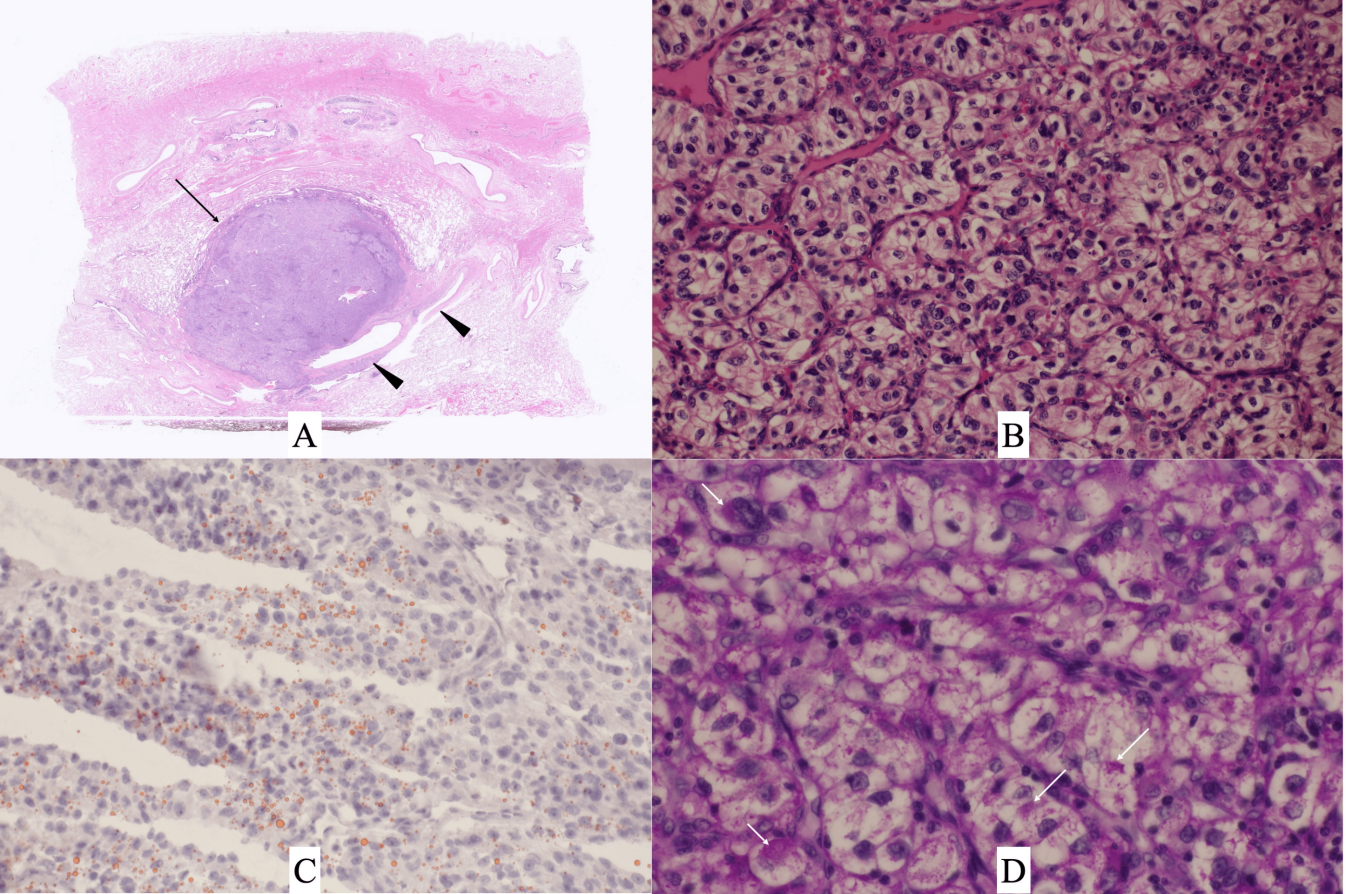
Pathological findings A. Low-magnification view showed a well-demarcated mass (arrow) adjacent to the pulmonary veins (arrowheads). B. Atypical cells with clear cytoplasm showed growth in an alveolar pattern without any intermingling of adipocytes (H.E. staining). C. Sudan staining showed micro lipid droplets (small yellow particles) on the cancer cells. D. PAS-positive granules were observed in the cytoplasm of cancer cells (arrows). PAS: Periodic acid-Schiff

Although PET findings were not strongly indicative of a metastatic lung tumor, rapid tumor growth made the patient undergo right lower lobectomy to avoid under treatment. The postoperative pathological study showed a well-circumscribed oval tumor, 20 mm in size, consisting of atypical cells with clear cytoplasm and densely growing in an expansive manner (Figures 3A, 3B). Immunostaining using paraffin-embedded tissue showed that the atypical cells had a Ki-67 labelling index of 17%, were positive for CA9 and PAX8, and were negative for CD10 and TTF-1. In addition, lipid droplets were observed on the tumor cells and periodic acid-Schiff (PAS)-positive granules were confirmed in the cytoplasm of the atypical cells (Figures 3C, 3D).

The patient has been well for 17 months after the re-operation of the metastatic lung tumor.

## Discussion

Thoracic surgeons often surgically treat metastatic lung tumors of various solid malignancies [7]. Many patients with metastatic lung tumors can get not only extended survival but also cure for the metastatic lung tumors through some kind of surgical treatment [8]. Especially, solitary metastatic lung tumors with favorable biology and a long disease-free interval are good candidates for surgical resection.

PET/CT can depict not only the size and shape of the lung lesions but also their metabolic characteristics. PET/CT, therefore, is extremely useful for diagnosing small lung lesions and often leads to early application of local treatment to the lung lesions [9]. Especially, the SUV max value of PET can play an important role in the decision of surgical application to small lung lesions.

Signet-ring cell carcinoma is one of the aggressive malignant tumors. It, however, is well known that PET often fails to show FDG accumulation even in large signet-ring cell carcinomas [10]. Abundant mucin in the cytoplasm of the signet-ring cell carcinomas leads to ostensible low SUV max values. It is very easy for oncologists to recognize this underestimation due to the characteristic pathological findings of signet-ring cell carcinomas.

The correlation between the clear cytoplasm and the intracytoplasmic substances in CCRCCs has been pathologically pointed out [11]. No diagnostic physicians, however, have pointed out the ostensible low values of SUV max in CCRCCs to date. PAS immunostaining clearly showed the presence of a certain amount of glycogen in the cancer cells. Unfortunately, lipid droplets were observed not in the cancer cell cytoplasm but just on the cancer cells in this case presumably due to the alcohol use during the formalin embedding. In short, intracytoplasmic lipids were eluted from the cytoplasm by alcohol use and observed as lipid droplets on cancer cells. The absence of adipocytes in the background tumor tissue highly supports this speculation. Taken together, there is a possibility that CCRCCs can be underestimated by PET.

In this case, more than one year had passed since the detection of the lung nodule to the lung surgery due to the diagnostic difficulty. Caution, therefore, should be taken when evaluating the metastatic CCRCC foci using PET. In fact, the SUV max value of metastatic lung tumor, similar in size to this case, from other solid malignancies (Figure 2B) is much higher than that observed in this case.

## Conclusions

Metastatic lung tumors from CCRCCs are oval or round in shape with distinct borders which are often difficult to be judged as malignant when detected in small sizes. PET can generally offer very important image findings on such situations in various solid malignancies. Oncologists, however, should note that the SUV max value of small metastatic lung tumors from CCRCCs is under-expressed due to the presence of lipids and glycogen in the cancer cell cytoplasm.
